# The Mississippi Valley Medical Association

**Published:** 1886-11

**Authors:** 


THE MISSISSIPPI VALLEY MEDICAL ASSOCIATION.

Since the last meeting of the Mississippi Valley Medical Association, medical journals in different parts of the country have expressed the opinion that the association was to be made for the west what, by some it is charged that "The Association of American Physicians" is designed to be for the east, viz., antagonistic to the American Medical Association. In view of the action taken by the Mississippi Valley Medical Association, at the Quincy meeting this year, it is difficult to understand how such an impression has been created. The Mississippi Valley Medical Association has been in existence (formerly under the name of the *Tri-State Medical Society*) for a number of years, and it is a working society. It has never had to do with medical politics, and at its last meeting it adopted the code of ethics of the American Medical Association as part of its constitution to remove the unjust impression regarding it, as shown at the last meeting of the American Medical Association at St. Louis. The society, it is true, embraces many members of the medical profession in the Mississippi Valley, but it in no sense attempts to antagonize the national association.

